# Heater Integrated Lab-on-a-Chip Device for Rapid HLA Alleles Amplification towards Prevention of Drug Hypersensitivity

**DOI:** 10.3390/s21103413

**Published:** 2021-05-13

**Authors:** Shah Mukim Uddin, Abkar Sayad, Jianxiong Chan, Duc Hau Huynh, Efstratios Skafidas, Patrick Kwan

**Affiliations:** 1 Department of Medicine, The University of Melbourne, Royal Melbourne Hospital, Melbourne, VIC 3050, Australia; suddin1@student.unimelb.edu.au (S.M.U.); jianxiong.chan@monash.edu (J.C.); duchau.huynh@gmail.com (D.H.H.); sskaf@unimelb.edu.au (E.S.); 2 Department of Neuroscience, Central Clinical School, Monash University, Melbourne, VIC 3004, Australia; abkar.sayad@monash.edu; 3 Department of Electrical and Electronic Engineering, Melbourne School of Engineering, The University of Melbourne, Melbourne, VIC 3010, Australia

**Keywords:** loop-mediated isothermal amplification, microfluidics, lab-on-a-chip, point-of-care diagnostics, pharmaco-genetics

## Abstract

HLA-B*15:02 screening before administering carbamazepine is recommended to prevent life-threatening hypersensitivity. However, the unavailability of a point-of-care device impedes this screening process. Our research group previously developed a two-step HLA-B*15:02 detection technique utilizing loop-mediated isothermal amplification (LAMP) on the tube, which requires two-stage device development to translate into a portable platform. Here, we report a heater-integrated lab-on-a-chip device for the LAMP amplification, which can rapidly detect HLA-B alleles colorimetrically. A gold-patterned micro-sized heater was integrated into a 3D-printed chip, allowing microfluidic pumping, valving, and incubation. The performance of the chip was tested with color dye. Then LAMP assay was conducted with human genomic DNA samples of known HLA-B genotypes in the LAMP-chip parallel with the tube assay. The LAMP-on-chip results showed a complete match with the LAMP-on-tube assay, demonstrating the detection system’s concurrence.

## 1. Introduction

Aromatic ring structured antiepileptic drugs (AEDs) such as phenytoin (PHT), lamotrigine (LTG), and carbamazepine (CBZ) are the most prevalent sources of severe cutaneous adverse drug reactions [1,2]. These range from benign to severe reactions, including drug reactions with eosinophilia, systemic symptoms (DRESS), acute generalized exanthematous pustulosis (AGEP), toxic epidermal necrolysis (TEN), and Stevens–Johnson syndrome (SJS) [3,4]. The incidence estimation of SJS/TEN ranges from 1 in 1000 to 10,000 drug exposures [5,6], and its mortality rate is as high as 35% [7,8]. Pharmacogenetic studies have discovered genetic associations between antiepileptic drugs-induced cutaneous adverse drug reactions and the human leukocyte antigen (HLA) alleles. Carbamazepine is an iminodibenzyl derivative that is extensively used in treating epilepsy and other indications such as neuralgia and bipolar affective disorder. Specifically, SJS/TEN induced by carbamazepine is strongly associated with HLA-B*15:02 in broad Asian ethnicities, including the Han Chinese and Thai population [9,10,11,12], but not in the Japanese [13] and European population [14]. Pre-treatment HLA genotyping is recommended to prevent carbamazepine induced SJS/TEN. However, several challenging factors need to be considered in the implementation of this recommendation [15].

Conventional methods to type HLA-B*15:02 include commercial polymerase chain reaction (PCR) with sequence-specific oligonucleotides (SSOP) or sequence-specific primer (SSP) [16], and sequence-based typing (SBT) [17]. These methods require expensive laboratory equipment, skilled laboratory personnel, and extensive processing time. Consequently, current methods pose barriers for prompt drug administration during or post-seizure, where prompt HLA-B*15:02 genotyping is necessary [18]. To overcome these barriers, our research group is developing a miniaturized point-of-care device for rapid genotyping of HLA-B*15:02. Crude blood samples were previously used in loop-mediated isothermal amplification (LAMP) reaction, which selectively amplifies selected areas in the HLA-B alleles. Afterward, the LAMP amplicon hybridizes with the DNA probes immobilized on the interdigitated electrode (IDE)-based biosensor surface that act as the mono-allelic determinant of an HLA-B*15:02 LAMP amplicon [19]. The probe hybridization process to complementary HLA-B*15:02 LAMP amplicons alters the biosensor’s electrical impedance to provide qualitative results.

Microfluidic devices are a basic element to develop the micro-total analysis systems (μTAS) or lab-on-a-chip device. These systems are classified as mechanical and non-mechanical based on the structure [20]. These systems are also classified as active or passive devices based on fluid flow techniques [21,22]. Active microfluidics [23,24] involve the motion or transportation of the biological samples applying an external source [25,26,27] or actuators [28]. On the other hand, the device’s physical configuration defines the system’s functionality in passive microfluidics. This device operates by the working fluid’s surface effects, such as surface tension, osmosis, pressure, capillary action, gravity-driven flow, vacuums, hydrostatic flow, and selective hydrophobic/hydrophilic control [29]. The structural complexity of the passive devices is relatively higher compared to the active device. Hence, the integration of passive microfluidics is challenging in point-of-care applications [30]. Microvalves and micro-pumps are the foundation of microfluidic systems.

Microvalves allow the regulation of liquid flow in a micro-channel by varying a macroscopic parameter or actuator. Microvalves’ functions include on-off switching, flow regulation, flow routing, fluid separation, and sealing biomolecule/particles in the incubation chamber. Such microvalves need to fulfill several requirements to integrate the DNA amplification process. Firstly, the valves need to withhold the pressure produced throughout the incubation period because of the sample evaporation and air expansion at high temperature. The valve needs to ensure the amplified sample’s confinement inside the reaction chamber without leakage flow during amplification. Secondly, the valve material needs to be chemically resilient because the valves will contact the LAMP solution. Multiple research groups have reviewed the range of valves with different working principles [31,32,33,34,35]. Among the valve’s variants, mechanical active microvalves are easier to develop and require less complicated microfluidic design. Mechanical active valves are designed utilizing the Bio-MEMS-based surface micromachining technologies, where mechanically movable membranes are coupled to magnetic, electric, thermal, and piezoelectric actuators. The micropump function in the microfluidic device is to pressurize the working liquid for flowing through the system. The fluid transport mechanism of the pressure-driven microfluidic device is based on pressure gradients. The mechanical micropump usually utilizes a physical actuator or moving parts such as oscillating membranes/diaphragm, piston, or turbines for delivering a persistent fluid volume. Multiple research groups have reviewed the micropump range with different working principles [25,28,31,32,33,36,37,38,39,40,41].

LAMP [42] technique operates at a constant temperature which reduces the requirements for the microfluidic feature. The typical operating temperature for LAMP is 60–65 °C, and the amplification time is 15–60 min. Hence, the LAMP device specification is relatively simpler than standard PCR, making this technique a promising DNA amplification alternative and ideal for point-of-care (POC) applications [43,44,45]. A critical step of the LAMP chip development is the device material selection considering an adverse effect, disposability, manufacturability, and cost-effectiveness. Different materials are utilized for microfluidic device construction, such as glass [46], silicon [47], polydimethylsiloxane (PDMS) [48,49,50], PMMA [51,52,53], polystyrene (PS) [54], polycarbonate (PC) [55], cyclic olefin polymer (COP) [56], and cyclic olefin copolymer (COC) [57]. PDMS is one of the most preferred polymeric materials for rapid prototyping of microfluidic devices, but it is expensive due to the photolithography requirement. Thermoplastic polymers such as PMMA, PC, PS, COC, and COP are widely utilized due to their exceptional chemical and physical properties. Glass is used due to its favorable optical and electrical properties. Silicon is used because of its good thermal conductivity, making it ideal for rapid heating and cooling. Several review articles critically analyzed the advantages and drawbacks of the currently developed microfluidic chips to apply the DNA amplification-based diagnosis process [20,43,44,58,59,60,61,62,63,64,65,66,67,68,69]. Different aspects affect the performances of the microfluidic devices incorporating LAMP amplification. Some of them are related to the assay’s miniaturization, others are related to the isothermal amplification methods. This concern encourages the researchers to develop new microfluidic devices integrated with simplified assays.

We previously developed a LAMP-IDE platform for HLA-B*15:02 genotyping, which requires a two-step process [19]. Firstly, HLA-B alleles amplification is performed using the LAMP technique on the tube. Secondly, LAMP amplicon hybridization on the developed biosensor surface occurs as a mono-allelic determinant of HLA-B*15:02. In that study, LAMP was performed in a tabletop thermocycler, and the detection process was performed with manual sample handling as a proof-of-concept. To transform this tabletop platform into a portable platform, a 2-step POC device development is required. This will require a microfluidic chip to perform the LAMP reaction and then integrate the previously developed biosensor into the LAMP chip. Here, we describe a LAMP-on-a-chip device integrating microfluidic operations and microheater towards developing a POC device for precision medicine.

## 2. Materials and Methods

### 2.1. Samples and LAMP Reaction

Purified human genomic DNA samples of known HLA-B genotypes were used to perform the LAMP reaction acquired from healthy donors (University of Melbourne ethics committee, ethics ID 1443204.4). The LAMP primer set was acquired from the previous study, which our research group designed to amplify the HLA-B gene exon 2 [19]. LAMP reagent contains 1XWarmStart^®^ Colorimetric LAMP Master Mix (New England Biolabs, Ipswich, Massachusetts), 1.6 µM inner primers (FIP and BIP), and 0.2 µM forward and reverse primers (F3 and B3). All primers utilized were desalted grade (Integrated DNA Technologies, Coralville, USA). A template volume of 1 μL was added with a concentration of 50 ng/μL in each reaction. A template of HLA-B*15:02/HLA B75 was used as a positive control and HLA-B*08:01/HLA B8 as a negative control. The blank negative control was the nuclease-free water. Each reaction was adjusted to a final volume of 12.5 μL using nuclease-free water. LAMP was conducted at 65 °C for 20 min utilizing a thermal cycler (Bio-Rad, Hercules, California). The LAMP reagents and DNA template were mixed on the tube before loading into the LAMP-chip. After the LAMP reaction, the positive control changes to yellow from its original pink color, whereas the negative color is unchanged.

### 2.2. Micro-Valve Design

**Figure 1:**
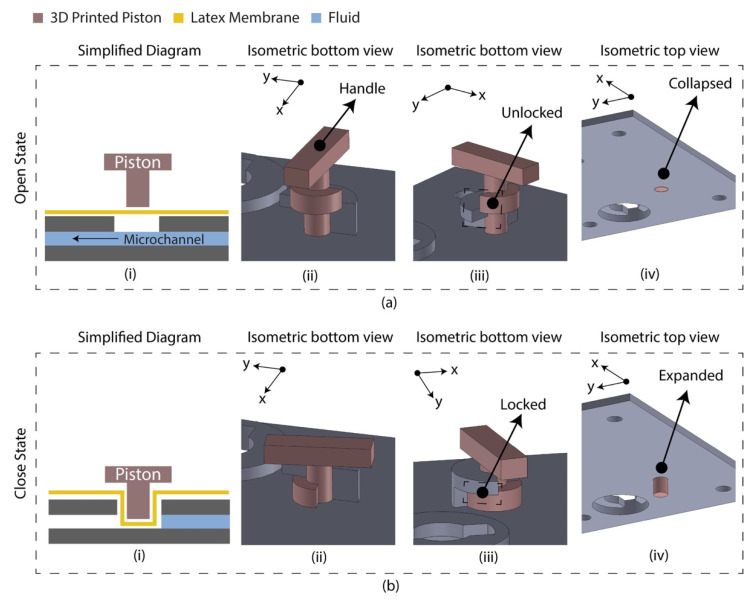
Latex membrane-based micro-valve design in two operating states. (**a**) Open state. The piston requires anticlockwise rotation (90°) and pulling to change state from close to open; (i) simplified diagram indicating the floated piston on the latex membrane allowing the fluid to flow; (ii) isometric view of the valve indicating the handle for manual control; (iii) isometric view indicating the unlocked/pulled condition; (iv) isometric view (isolated) indicating the collapsed condition of the piston. (**b**) Closed state. The piston requires pushing and clockwise rotation (90°) to change state from open to close; (i) simplified diagram indicating the piston blocked the microchannel deforming the latex membrane; (ii) isometric view of the valve; (iii) isometric view indicating the locked/pushed condition; (iv) isometric view (isolated) indicating the expanded condition of the piston.

The diaphragm-based active microfluidic system utilizes a reciprocating membrane actuated by a physical actuator to control the microfluidic operation. A latex membrane-based microfluidic valve was designed using 3D-printed parts manually controlled by the piston. The 3D-printed parts and the latex membrane were bonded using a pressure-sensitive adhesive (PSA). Figure 1 depicts the schematic of the developed microfluidic valve mechanism. At an open state, the valve piston rests on the latex membrane, allowing the fluid to flow through the microchannel. At a closed sate, the valve piston is pushed down to the microchannel by deforming the latex membrane, which blocks the fluid flow. This valve’s mechanical structure allows the piston to unlock/lock the position at an open/close state.

### 2.3. Micro-Pump Design

**Figure 2:**
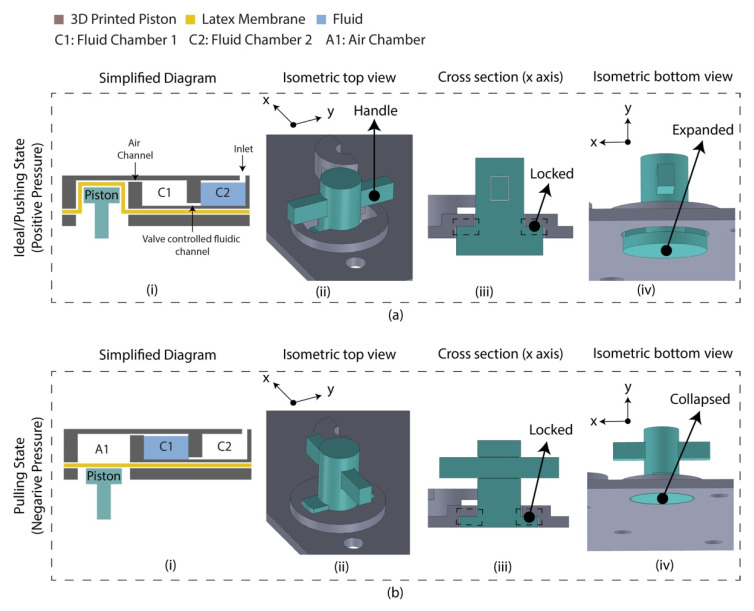
Latex membrane-based micro-pump design in two operating states. (**a**) Ideal/pushing state. The piston requires push action and anticlockwise rotation (90°) to change the state from pulled to pushed; (i) simplified diagram indicating the piston placed inside the air chamber by deforming the latex membrane. The chip was assembled in this state which is referred to as an ‘ideal state’; (ii) isometric view of the micro-pump indicating the handle for manual control; (iii) cross section along the *x* axis indicating the locked condition; (iv) isometric view (isolated) indicating the expanded condition of the piston. (**b**) Pulling state. The piston requires clockwise rotation (90°) and pull action to change the state from pushed to pull; (i) simplified diagram indicating the floated piston on the latex membrane; (ii) isometric view of the micro-pump; (iii) cross section along the *x* axis indicating the locked condition; (iv) isometric view (isolated) indicating the collapsed condition of the piston.

A diaphragm-based active microfluidic pump was designed using 3D-printed parts, which were bonded using PSA. Reciprocation of the membrane in a mechanical micropump occurs utilizing a physical actuator that generates the required pressure deviation to pump the fluid. Figure 2 depicts the schematic of the developed microfluidic pump. This micro-pump utilizes a manually controlled piston placed on the air chamber, creating positive/negative pressure into the sample chamber. Based on the Hagen–Poiseuille equation, the pressure difference between the two ends of a channel is Δ*p*,

$$\Delta p=\frac{8\mu LQ}{\pi R^{4}}$$

where *L* is the channel’s length, *R* is the channel’s radius, *μ* is the reagent’s dynamic viscosity, and *Q* is the volumetric flow rate. Considering the flow rate was controlled manually by the finger-controlled piston, the channel’s dimension is constant, and the pressure required to transport the fluid between C1 and C2 chamber is defined by the A1 chamber’s volume for the reagents. The A1 chamber volume was optimized to 2.5 times of the fluidic chambers, experimentally. At an idle state, the piston was placed inside the air chamber (A1) by deforming the latex membrane where the fluidic is located in chamber 2 (C2), and chamber 1 (C1) is filled with air. By pulling the piston downward, a negative pressure is created in the A1 chamber, which leads to moving air from C1 to A1 and the sample from C2 to C1. This state is defined as a pulling state. The piston needs to be moved reversely to transport the liquid from C1 to C2. This state is defined as a pushing state. This micro-pump’s mechanical structure allows the piston to lock the position in a pulling/pushing state.

### 2.4. Micro-Heater Design

**Figure 3:**
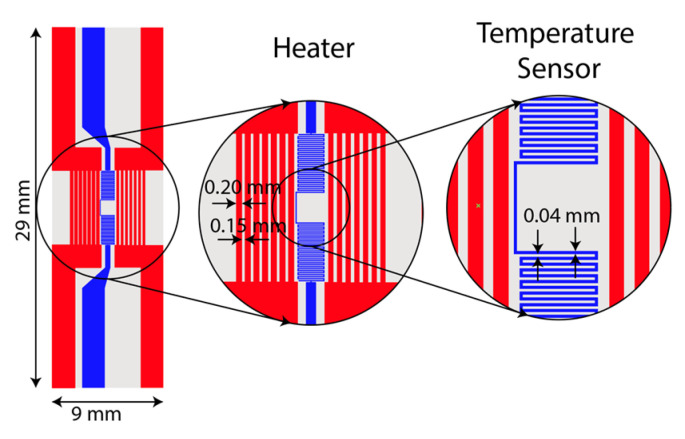
Photograph and schematic of the integrated heater and temperature sensor on a glass substrate.

A resistive micro-heater was fabricated to integrate into the LAMP-chip. Figure 3 depicts the schematic and photograph of the fabricated heater and sensor. The micro-heater has two sets of electrodes, (a) a heating electrode and (b) a temperature sensing electrode. The heating electrode has 14 parallel fingers (finger length: 6 mm, finger width: 200 µm, finger gap: 150 µm). The temperature sensing electrode is a conductor line of zigzag pattern (length 70 mm, width 40 µm, and gap 40 µm). These electrodes are made with multiple chemical elements (5nm Ti/100 nm Au/5 nm Ti/25 nm Si_2_O). The fabrication process was reported in one of our previous articles [19].

### 2.5. LAMP-Chip Construction

**Figure 4:**
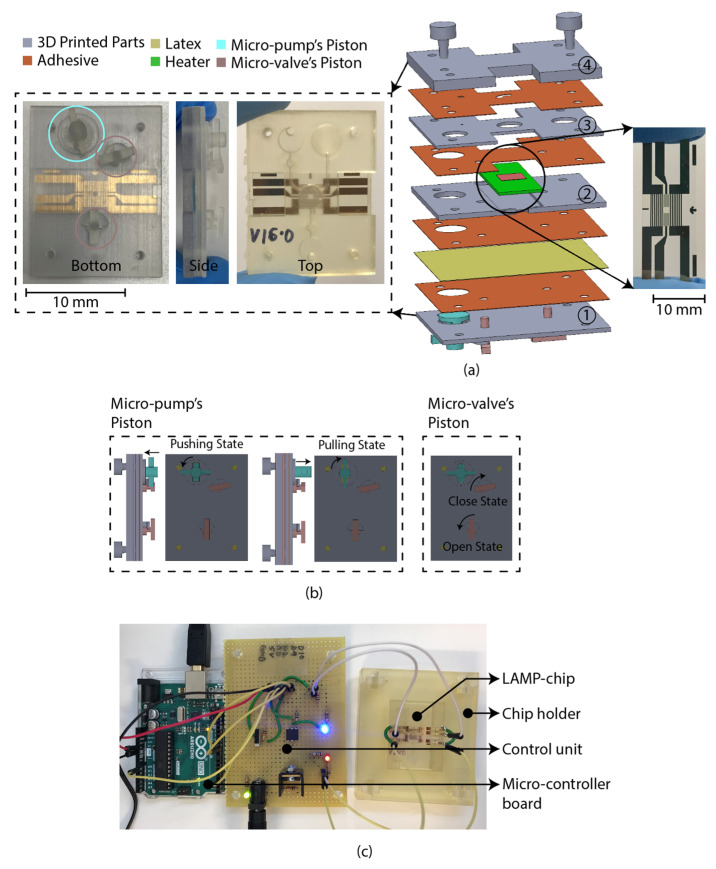
LAMP-chip (**a**) photo and exploded 3D schematic. The primary function of the four 3D-printed parts: (1) supports the pistons, (2) supports the heater, (3) chamber feature, (4) inlet-outlet feature. The latex membrane supports the functionality of the micro-pump and the micro-valve. (**b**) Micro-pump’s push-pull state and micro-valve’s open-close state. Anticlockwise rotation and push action are required to create positive pressure (pushing state). Clockwise rotation and pulling action are required to create negative pressure (pulling state). (**c**) Experimental setup consists of LAMP-chip with the chip holder and control circuit board.

The LAMP-chip was constructed with multiple 3D-printed parts, PSA, latex membrane, and micro-heater. The overall size of the LAMP-chips is length 4 cm × width 3 cm × thickness 1 cm. Figure 4a depicts the 3D schematic (exploded view) and a photograph of the developed LAMP-chip. This chip facilitates two microfluidic features (i.e., micro-pump and micro-valve) to perform one LAMP reaction per device. Figure 4b depicts the piston’s action of the micro-pump and micro-valve, which are based on the working principle explained in Section 2.2 and Section 2.3. The parts of the LAMP-chip were 3D printed using Objet Eden 260V printer with RGD720 biocompatible material. The PSA cut-out was prepared using the Roland CAMM-1 GS-24 desktop cutter. The layers of the LAMP-chip were aligned using a metallic pin set. A systematic layer alignment sequence was followed to avoid micro-heater damage. Afterward, high pressure was imposed on the chip using a manual press machine to bond them properly. A proportional–integral–derivative controller (PID)-based circuitry and firmware was developed to operate the micro-heater. The micro-heater was calibrated using a commercial IR thermometer (manufacturer part no. Fluke 64 MAX). The control circuitry could be powered with a 5-volt portable battery, and a user interface could be developed to operate with a mobile phone. In this study, the experimental setup was controlled with a computer. Figure 4b shows the experimental setup, which has two major parts, (a) PID control circuitry with Arduino microcontroller board, and (b) LAMP-chip on a 3D-printed holder.

## 3. Results

The performance test of the LAMP-chip was conducted in two phases. First, the LAMP-chip was tested with the color dye to assess the microfluidic operation, incubation chamber sealing, and micro-heater performance. Second, the LAMP assay with purified human genomic DNA samples and visual detection was performed on-chip and tube.

### 3.1. Performance Test

**Figure 5:**
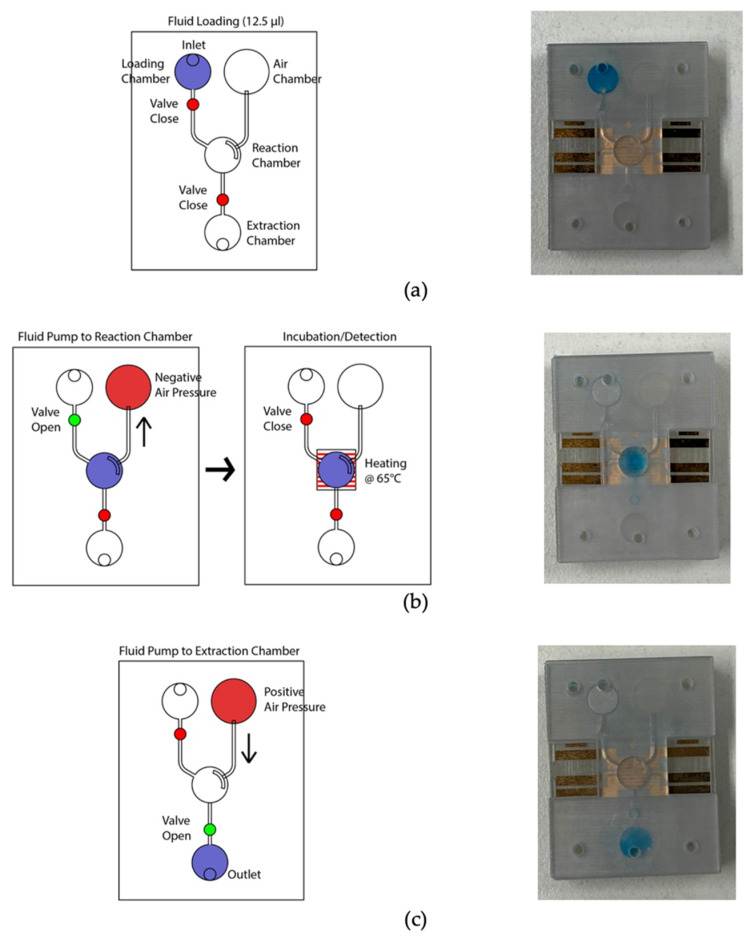
Photograph of the LAMP-chip in operation in line with the corresponding schematics. (**a**) Sample loading state, (**b**) sample transfer from loading chamber to amplification chamber, followed by the sample heating state, and (**c**) sample extraction state.

**Figure 6:**
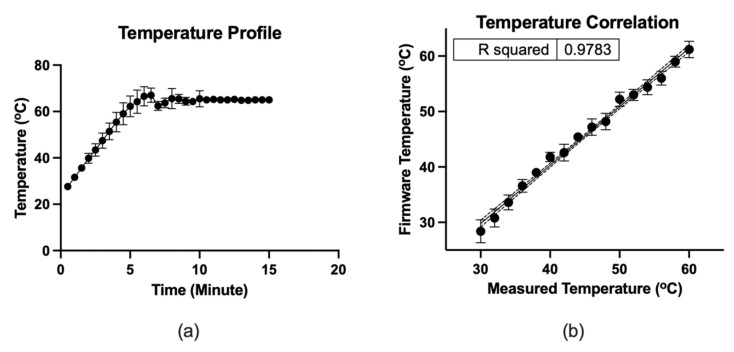
(**a**) Temperature profile of the micro-heater. (**b**) Correlation between the firmware readout and the measured thermometer by IR thermometer, where *n* = 5. The dots indicate the mean, and the error bars represent the standard deviation.

Figure 5 depicts the microfluidic operation. At the initial condition, all valves were closed, and the micro-pump was in a pushing state. Firstly, the valves were closed, and 12.5 µL color dye was loaded into the loading chamber using a pipette (Figure 5a). The connecting valve was opened, and negative air pressure was initiated by pulling the air chamber’s piston to transfer the liquid to the amplification chamber. Consequently, the fluid moved to the amplification chamber from the loading chamber (Figure 5b). At this stage, both valves were closed to seal the reaction chamber. The micro-heater was activated at 65 °C using firmware and heated for 20 min. The firmware analyzed the temperature sensing electrodes’ resistance, which corresponds to the firmware temperature and adjusted the heating electrode’s power in real-time to stabilize the temperature at 65 °C. In addition to the firmware, the heating chamber’s temperature was recorded using the IR thermometer for comparison. Figure 6 shows the micro-heater’s temperature profile and correlation (R^2^ = 0.9783) between the firmware readout and the reference thermometer readout, where *n* = 5. The micro-heater took about 5 min to reach 65 °C. Sample leakage in the amplification chamber was not found in the LAMP-chips. The fluid was transferred from the amplification chamber to the extraction chamber by opening the connecting valve and initiating a positive pressure at the air chamber to extract the amplified sample (Figure 5c). The sample extraction from the extraction chamber was performed using a pipette.

### 3.2. LAMP Assay

**Figure 7:**
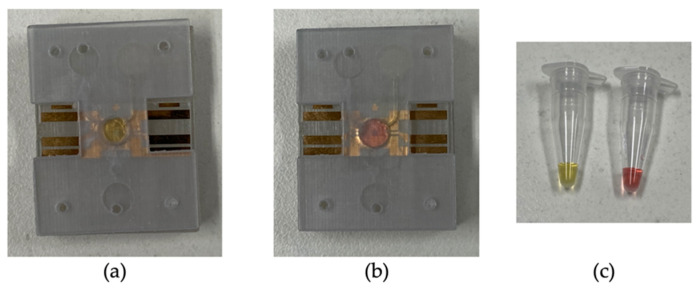
Representative photographs of the LAMP-chip and LAMP-tube after the amplification. (**a**) Positive control on LAMP-chip, (**b**) negative control on LAMP-chip, and (**c**) positive and negative control on LAMP-tube.

A parallel assay was performed on the LAMP-chip and tube. Table 1 indicates the quantity of each kind with the corresponding controls. The colorimetric master mix, primers, and the template were mixed in a tube before loading into the LAMP-chip. Afterwards, the microfluidic operation was executed following the sequence detailed in Section 3.1, and the tube-based LAMP assay was performed in the thermal cycler. In the LAMP-chip, the color started to change about 20 min into the incubation period, and the differentiation became more evident after 25 min. The color of the positive controls changed to yellow from the original pink color, whereas the negative controls remained unchanged. The color of the positive and negative LAMP amplicons was distinguishable in ambient lighting conditions for both platforms. Figure 7 shows the color differentiation of positive and negative control for both platforms. The detection result was found to be the same on both platforms. Non-specific amplification in the off- and on-chip tests was not observed, in line with our previous report [19].

## 4. Discussion

The micro-valve and micro-pump mechanism required to develop the LAMP-chip can be adopted from different microfluidic operating principles [70,71,72]. Each of these principles has its unique advantages and limitations. There are no standardized metrics of performance for microvalves and micropumps. They are different in materials, cost, fabrication, portability, biocompatibility, and reusability. The particular properties that make a micro-valve or micro-pump design appealing for one specific application may also exhibit relatively less efficiency in other applications. Hence, these properties should be considered as a whole to determine the design suitability for a particular biomedical application. A range of technical information is available for a microfluidic system which still needs to be incorporated into a tangible device. The commercialization of microfluidic technologies for biomedical applications is still in infancy.

In this study, 3D printing technology was exploited to develop the microfluidic device for its unique ability to construct a complex functional structure which would be challenging for another microfluidic platform. Three-dimensional printing is an effective alternative method to fabricate structurally vigorous microfluidic devices [73,74]. The 3D-printed microfluidics’ primary limitation is the printing resolution, which is limited to 200 μm [74], whereas the critical dimension for the developed LAMP-chip is 500 μm, in this study. Rogers et al. [75] demonstrated a 3D-printed valve for a microchannel of 350 μm diameter and limited to opaque material, which is not suitable for optical detection. Wang et al. [76] demonstrated a 3D-printed microfluidic system utilizing a desktop syringe pump with a flow rate of 15 μL/min. Anthony et al. [77] demonstrated a stereo-lithographically printed microfluidic system with optically clear biocompatible plastic, where three-port plug-in style valves were unitized to actuate the microfluid operation. The overall performance of the micro-pump and the micro-valve is similar to other microfluidic systems based on syringe pumps or off-chip electronic valves. The desktop syringe pump-based microfluidic system could be impractical for POCT devices, and the membrane-based microfluidic system would be appropriate. We foresee that this first-generation prototype can be further developed to automate the microfluidic actuation, appealing for POCT devices.

In the past decade, research exploring the potentials to translate the LAMP-on-tube technique into LAMP-on-chip due to its unique advantage of single amplification temperature and low reagent cost [58]. Several lab-on-a-disk-based LAMP devices have been developed integrating preloaded reagent mixes and detection agents [52,78,79,80]. Researchers have explored paper-based devices for their robustness, cost-effectiveness, and user-friendliness [81,82]. A magnetic bead-based LAMP system was designed to detect methicillin-resistant Staphylococcus aureus (MRSA), integrating nucleic acid extraction and temperature control [83]. Liang et al. developed a LAMP-based thermal digital microfluidic device called Lamport to detect Trypanosoma brucei, which required SYBR Green I after the amplification for endpoint detection [84]. Multiple LAMP endpoint detection methods have been developed in the past 20 years, such as naked-eye observation of precipitation [85], colorimetric detection [86,87], gel electrophoresis [88], and biosensor-based electrochemical methods [19,89]. A substantial report is available to perform the LAMP reaction on-chip in various microfluidic platforms, but the POC device development for precision medicine such as HLA genotyping is still in infancy.

In this study, a LAMP-chip was developed to amplify the HLA-B alleles as the first step of the two-step POC device development process. A diaphragm-based microfluidic system was developed to transport the sample into the reaction chamber and seal the chamber during the high-temperature amplification. In this system, a finger-controlled piston was utilized to execute the microfluidic operation, which can be further developed in the future with an electronic mechanism to automate the operation. A gold patterned heating unit was integrated with the incubation chamber to conduct the LAMP reaction at 65 °C. A two-stage test was performed in this study. In stage 1, the microfluidic system was tested with color dye, indicating successful fluid flow and chamber sealing during incubation. The heating unit showed a correlation of R^2^ = 0.9783 with a reference thermometer during the temperature ramp and maintained the temperature at the setpoint with a tolerance of ±1 °C. In stage 2, a colorimetric LAMP master mix was utilized to assess the amplification outcome in a chip, which is in line with tube assay. This micro-heater unit may also be combined with an interdigitated electrode (IDE) biosensor [19] in the future.

## 5. Conclusions

We have developed a LAMP-chip to amplify the HLA-B alleles, which is the first part of the two-step HLA-B*15:02 detection process. Previously, we reported the LAMP detection technique for HLA genotyping in a tube-based platform, whereas here, we improved the technique to perform in the chip for point-of-care application. The chip is featured with microfluidic operations (i.e., micro-pump, micro-valve, and micro-heater) to perform LAMP amplification in a portable manner. The microfluidic performance was tested with color dye, demonstrating microfluidic transportation capabilities, strong temperature correlation (R^2^ = 0.9783) with a reference thermometer, and temperature stabilization (setpoint 65 °C) at 10 min. The micro-valve mechanism can contain the amplicons in the high-pressure amplification chamber. The color change is visible in ambient lighting conditions after the 25-min on-chip amplification. The LAMP-on-chip results showed a complete match with the LAMP-on-tube assay, demonstrating the detection system’s concordance. This LAMP-chip can be adapted for another nucleic acid amplification-based biomarker detection when prompt genetic information is required for clinical decisions.

## Figures and Tables

**Table 1:** LAMP assays on chip and in tube.

Assay Platform	Control Type	Template	Quantity (pcs)
LAMP-chip	Positive	HLA-B*15:02/HLA B75	5
	Negative	HLA-B*08:01/HLA B8	2
Tube	Positive	HLA-B*15:02/HLA B75	2
	Negative	HLA-B*08:01/HLA B8	2
		Nuclease-free water	1

## Data Availability

Not applicable.
